# Success and complications by team composition for prehospital paediatric intubation: a systematic review and meta-analysis

**DOI:** 10.1186/s13054-020-02865-y

**Published:** 2020-04-15

**Authors:** Alan A. Garner, Nicholas Bennett, Andrew Weatherall, Anna Lee

**Affiliations:** 1 CareFlight Australia, 4 Barden St, Northmead, NSW 2152 Australia; 2 The University of Sydney, Sydney, Australia; 3 Division of Paediatrics and Child Health, The University of Sydney, Sydney, Australia; 4 Department of Anaesthesia and Intensive Care, The Chinese University of Hong Kong, Shatin, Hong Kong; 5 Hong Kong Branch of The Chinese Cochrane Centre, The Jockey Club School of Public Health and Primary Care, Faculty of Medicine, The Chinese University of Hong Kong, Shatin, Hong Kong

**Keywords:** Airway management, Child, Complications, Emergency medical services, Intubation

## Abstract

**Background:**

Clinical team composition for prehospital paediatric intubation may affect success and complication rates. We performed a systematic review and meta-analysis to determine the success and complication rates by type of clinical team.

**Methods:**

We searched MEDLINE, EMBASE, and CINAHL for interventional and observational studies describing prehospital intubation attempts in children with overall success, first-pass success, and complication rates. Eligible studies, data extraction, and assessment of risk of bias were assessed independently by two reviewers. We performed a random-effects meta-analysis of proportions.

**Results:**

Forty studies (1989 to 2019) described three types of clinical teams: non-physician teams with no relaxants (22 studies, *n* = 7602), non-physician teams with relaxants (12 studies, *n* = 2185), and physician teams with relaxants (12 studies, *n* = 1780). Twenty-two (*n* = 3747) and 18 (*n* = 7820) studies were at low and moderate risk of bias, respectively. Non-physician teams without relaxants had lower overall intubation success rate (72%, 95% CI 67–76%) than non-physician teams with relaxants (95%, 95% CI 93–98%) and physician teams (99%, 95% CI 97–100%). Physician teams had higher first-pass success rate (91%, 95% CI 86–95%) than non-physicians with (75%, 95% CI 69–81%) and without (55%, 95% CI 48–63%) relaxants. Overall airway complication rate was lower in physician teams (10%, 95% CI 3–22%) than non-physicians with (30%, 95% CI 23–38%) and without (39%, 95% CI 28–51%) relaxants.

**Conclusion:**

Physician teams had higher rates of intubation success and lower rates of overall airway complications than other team types. Physician prehospital teams should be utilised wherever practicable for critically ill children requiring prehospital intubation.

## Background

Airway management is a critical component of prehospital care for severely ill and injured children. Airway management is arguably even more important in children than in adults, as cardiac arrest is more likely to be hypoxic in origin and therefore amenable to airway and ventilation intervention. As hypoxia correction is a time critical intervention, an emergency medical service (EMS) system must be able to provide airway management as early as possible, preferably at the incident scene.

Intubation is generally considered to be the gold standard for airway management in the critically ill and injured. Children however typically comprise only about 5% of total EMS cases [1–3], and those requiring intubation vary from 0.1% of all EMS responses [3, 4] to approximately 5% of paediatric cases when advanced intervention teams are selectively utilised [1, 2]. Success rates are also reported to be lower in children and the complication rate higher [5, 6]. Traditionally, ground EMS systems have intubated children without muscle relaxants, but many systems are introducing relaxants into their clinical protocols with the expectation that overall success rates would improve and that intubation could be offered for a wider range of pathologies. There are also recent reports that physician staffed helicopter EMS (PS-HEMS) may produce particularly high procedural success with low complication rates [7–10].

The purpose of this study was to systematically review the available literature and perform a meta-analysis to determine whether there exists an association between type of prehospital team and intubation success and complication rates.

We hypothesised that utilisation of muscle relaxants by non-physician teams would improve procedural success in prehospital paediatric intubation over teams without relaxant access and that the greater experience and training of physician teams might produce further performance gains above those associated with relaxant access for non-physician teams.

## Methods

The systematic review was conducted and reported in accordance with the PRISMA (Preferred Reporting Items for Systematic Reviews and Meta-Analyses) Guidelines [11].

### Data sources and literature search strategy

We created search strategies for the concepts of ‘intubation’, ‘prehospital’, and ‘paediatric’ using a combination of standardised terms and keywords drawn from indices, thesauri, and on-topic articles (Supplementary eAppendix) in consultation with a medical librarian. The electronic databases Ovid MEDLINE, EMBASE, and CINAHL were searched from database inception to November 11, 2019. Additionally, we conducted a manual search of reference lists of included and other relevant articles. All articles were reviewed for inclusion by two independent reviewers (AG and NB). Any discrepancies were resolved by consensus with a third reviewer (AW).

### Study selection

Interventional and/or observational studies were eligible for inclusion if they reported data on the success, first-pass success, and/or complication rates of prehospital paediatric intubation attempts. Studies that did not separately report the number of patients in whom intubation was attempted were excluded from analysis, as were abstract-only and grey literature reports. There was no language restriction.

### Data extraction

Successful intubation, first-pass success rate, and complication rates were extracted from the included articles by two independent investigators (AG and NB). Where there was discrepancy, a third author (AL) adjudicated. We also extracted data about authors, publication year, study location, setting, professional background of team members, availability of muscle relaxants, and participant characteristics by inclusion age and intubation indication. For each study, the team composition (exposure variable) was classified into three groups: non-physicians with no relaxants, non-physicians with relaxants, and physicians with relaxants. We made no contact with authors for missing data as many studies were old.

### Outcomes

The primary outcomes were the proportions of overall intubation and first look success rates. Secondary outcomes included the rate of intubation complications, specifically unrecognised oesophageal and endobronchial intubation, three or more attempts at intubation, hypoxia, or aspiration.

### Assessment of study quality

The criteria used by Fouche and colleagues [12] were used to evaluate study quality. The checklist consists of 8 items that assess external and internal validity through 4 domains: selection bias, non-response bias, measurement bias, and bias related to the analysis, with each item graded as low or high [12]. The overall risk of bias for the study was rated ‘low’ if 7 or more domains were rated low, ‘moderate’ if 4 to 6 domains were rated low, and ‘high’ if 1 to 3 domains were rated low [12]. Each included study was assessed by AL and reviewed by AG.

### Data synthesis and statistical analysis

We used the macro ‘metaprop_one’ in STATA 16.0 (StataCorp, College Station, TX) to pool proportions using the Freeman-Tukey double arcsine option to ensure that the confidence intervals around the estimates did not fall outside 0 or 1 with stable variances [13]. We used a logistic-normal random-effects model [13] and assessed the heterogeneity as low, moderate, and high using *I*^2^ values of 25%, 50%, and 75% [14]. We performed subgroup analyses by team composition a priori to explain heterogeneity and conducted a sensitivity analysis on low risk of bias trials to estimate the robustness of primary outcome results. Meta-regression with robust variance estimates (to take into account within-study correlation between different team types) was used to explore differences in the primary outcomes by team composition subgroups over time (year of publication) [15]. As there were large variations in the clinical population (mixed, trauma, head injury, and arrested) studied, subgroup meta-analyses by team composition were also performed for the overall intubation rate, first-pass success rate, and overall airway complication rate. We did not assess publication bias with a funnel plot as it has been shown to be problematic in meta-analysis of proportions [16].

## Results

### Search results

**Fig. 1:**
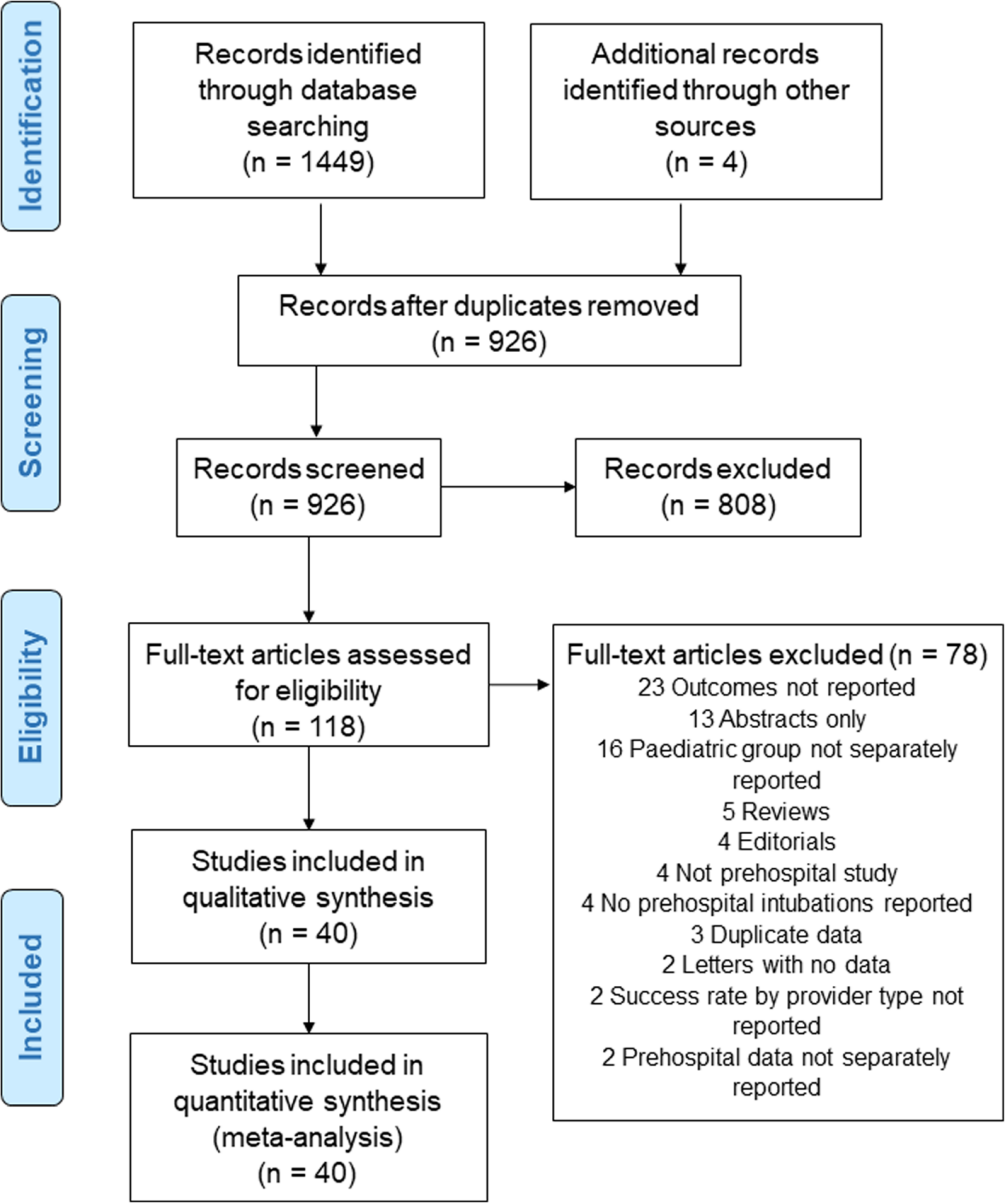
Systematic review flow chart

The search strategy yielded 40 eligible studies included in the analysis (Fig. 1). The characteristics of 40 included studies involving 11,567 children are shown in Table 1 [1, 3, 5–10, 17–48]. Fifteen studies (8201 participants) were published from 2015 onwards. The median (IQR) sample size of the studies was 86 (36 to 270). Twenty-six studies were published in the USA, five in Australia, two in the Netherlands, and one each in Belgium, Denmark, Finland, France, Germany, Switzerland, and the UK from 1989 to 2019.

**Table 1:** Characteristics of the included studies. Oesophageal intubation refers specifically to unrecognised oesophageal intubation. All physician teams utilised muscle relaxants

Author, year	Study country	Inclusion	No. of children	Group	Transport mode	Outcomes assessed	Overall risk of bias
Aijian 1989 [17]	USA	< 19 years, arrested patients only	28	Non-physician, no relaxants	Road	Overall intubation success, overall airway complication, oesophageal intubation, endobronchial intubation	Moderate
Andrew 2015 [18]	Australia	< 16 years	16	Non-physician, with relaxants	Helicopter	Overall and first look success rate, multiple attempts	Low
Babl 2001 [19]	USA	< 19 years	15	Non-physician, no relaxants	Road	Overall intubation success rate	Low
Baker 2009 [20]	USA	< 18 years	148	Non-physician, no relaxants	Road	Overall and first look success rate, overall complication rate	Moderate
Bankole 2011 [6]	USA	< 13 years, traumatic head injury only	31	Non-physician, no relaxants	Helicopter and road	Overall success and airway complication rate, oesophageal intubation, multiple attempts	Low
Boswell 1995 [21]	USA	< 14 years, unconscious trauma patients transported by helicopter only	58	Non-physician, no relaxants	Helicopter	Overall intubation success rate	Moderate
Brownstein 1996 [22]	USA	< 16 years, successful field intubations only	355	Non-physician, with relaxants	Road	Overall complication, oesophageal and endobronchial intubation rate, aspiration rate	Moderate
Burns 2017 [10]	Australia	< 16 years	82	Physician	Helicopter and road	Overall intubation and first look success, overall airway complication, oesophageal and endobronchial intubation, hypoxia, aspiration, multiple attempts	Low
Burton 2003 [23]	USA	< 13 years	137	Non-physician, no relaxants	Road	Overall intubation success rate	Low
Carlson 2015 [3]	USA	< 18 years	3049	Non-physician, no relaxants	Helicopter and road	Overall intubation success rate	Moderate
Demaret 2016 [24]	Belgium	< 16 years	353	Physician	Road	Overall intubation and first look success	Low
Dyson 2017 [25]	Australia	< 15 years	434	Non-physician, with relaxants	Road	Overall intubation and first look success	Low
Ehrlich 2004 [26]	USA	< 19 years, trauma only	59	Non-physician, with relaxants	Helicopter and road	First look success, overall airway complication, oesophageal and endobronchial intubation, aspiration rate	Moderate
Eich 2009 [1]	Germany	< 14 years	82, 58 reported in detail	Physician	Helicopter and road	Overall intubation and first look success, overall airway complication, oesophageal and endobronchial intubation, multiple attempts	Moderate
Garner 2019 [7]	Australia	< 16 years	(a) 7, (b) 61	(a) Non-physician, no relaxants; (b) physician	(a) Road, (b) helicopter	Overall intubation and first look success, overall airway complication, oesophageal and endobronchial intubation, hypoxia, aspiration rate, multiple attempts	Low
Garza 2005 [5]	USA	< 16 years, arrested patients only	86	Non-physician, no relaxants	Road	Overall intubation success rate	Moderate
Gausche 2000 [27]	USA	< 12 years	324	Non-physician, no relaxants	Road	Overall intubation success rate, overall airway complication, oesophageal and endobronchial intubation rate	Low
Gerritse 2010 [8]	Netherlands	< 16 years	(a) 86, (b) 214	(a) Non-physician, no relaxants; (b) physician	(a) Helicopter, (b) road	Overall intubation success, overall airway complication, oesophageal intubation rate	Low
Hansen 2015 [28]	USA	< 18 years	(a) 2444, (b) 408	(a) Non-physician, no relaxants; (b) non-physician, with relaxants	Helicopter and road	Overall intubation success, oesophageal intubation rate	Moderate
Hansen 2018 [29]	USA	< 18 years, transported cardiac arrests only	35	Non-physician, no relaxants	Road	Overall intubation success, multiple attempt rate	Low
Harrison 2004 [30]	USA	< 13 years	143	Non-physician, with relaxants	Helicopter and road	Overall intubation and first look success, oesophageal intubation, hypoxia, multiple attempts	Moderate
Heschl 2018 [31]	Australia	< 15 years	87	Non-physician, with relaxants	Helicopter	Overall intubation and first look success, oesophageal intubation, multiple attempts	Moderate
Jarvis 2019 [32]	USA	< 15 years	(a) 406, (b) 49	(a) Non-physician, no relaxants; (b) non-physician, with relaxants	Helicopter and road	First look success	Moderate
Kumar 1997 [33]	USA	< 16 years, arrested patients only	39	Non-physician, no relaxants	Road	Overall intubation success rate	Moderate
Lavery 1992 [34]	USA	< 19 years, trauma only	14	Non-physician, no relaxants	Road	Overall intubation success rate	Moderate
Losek 1989 [35]	USA	< 19 years	63	Non-physician, no relaxants	Road	Overall intubation and first look success, multiple attempts	Low
Losek 1994 [36]	USA	< 19 years	193	Non-physician, no relaxants	Road	Overall intubation success rate	Low
Martinon 2011 [37]	France	2–15 years, unintentional traumatic brain injury only	(a) 188, (b) 108	(a) Physician before guideline; (b) physician after guidelines	Road	Overall success rate, overall airway complication rates, hypoxia	Moderate
Moors 2018 [38]	Netherlands	< 17 years	(a) 79, (b) 103	(a) Non-physician, no relaxants; (b) physician	(a) Road, (b) helicopter	Overall intubation success, oesophageal and endobronchial intubation	Low
Nakayama 1990 [39]	USA	Trauma admissions to a paediatric trauma centre	14	Non-physician, no relaxants	Road	Overall intubation success, overall airway complication rate, oesophageal and endobronchial intubation rates	Moderate
Nevin 2014 [40]	UK	< 16 years, trauma only	315	Physician	Helicopter and road	Overall intubation success, oesophageal intubation rate, multiple attempts	Low
Paul 1999 [41]	USA	< 13 years, trauma only	6	Paramedic	Road	Overall intubation success rate	Moderate
Pointer 1989 [42]	USA	< 15 years	36	Non-physician, no relaxants	Road	Overall intubation and first look success, overall airway complication, oesophageal and endobronchial intubation, multiple attempts	Low
Prekker 2016 [43]	USA	< 13 years	299	Non-physician, with relaxants	Road	Overall intubation and first look success, overall airway complication, oesophageal and endobronchial intubation, aspiration rate, multiple attempts	Low
Schmidt 2016 [9]	Switzerland	< 17 years	215	Physician	Helicopter	Overall intubation and first look success, oesophageal intubation	Low
Simons 2017 [44]	Finland	< 16 years	34	Physician	Helicopter and road	Oesophageal and endobronchial intubation rate, aspiration rate	Moderate
Sing 1996 [45]	USA	< 18 years, relaxant assisted cases only	40	Non-physician, with relaxants	Helicopter	Overall intubation and first look success, overall airway complication, oesophageal and endobronchial intubation, hypoxia, aspiration rate	Low
Tarpgaard 2015 [46]	Denmark	< 16 years	25	Physician	Helicopter and road	Overall intubation and first look success, overall airway complication, oesophageal intubation, hypoxia, aspiration rate	Low
Tollefsen 2013 [47]	USA	< 15 years	260	Non-physician, with relaxants	Helicopter	Overall intubation and first look success, oesophageal intubation, multiple attempts	Low
Vilke 2002 [48]	USA	< 15 years	324	Non-physician, no relaxants	Helicopter and road	Overall intubation success, and oesophageal intubation	Low

Five studies [7, 8, 28, 32, 38] compared outcomes between different intubator groups, and one study [37] described outcomes before and after implementation of national guidelines. Of the 46 described team compositions, 22 studies utilised non-physicians with no relaxants (*n* = 7602), 12 utilised non-physicians with relaxants (*n* = 2185), and 12 utilised physicians, all with relaxants (*n* = 1780). Studies published before 2010 were mainly non-physicians with no relaxants (15/20). Since 2010, 11 of 12 studies involved physicians. Mode of transportation was road (24/46), helicopter (10/46), and both road and helicopter (12/46).

### Quality assessment

**Fig. 2:**
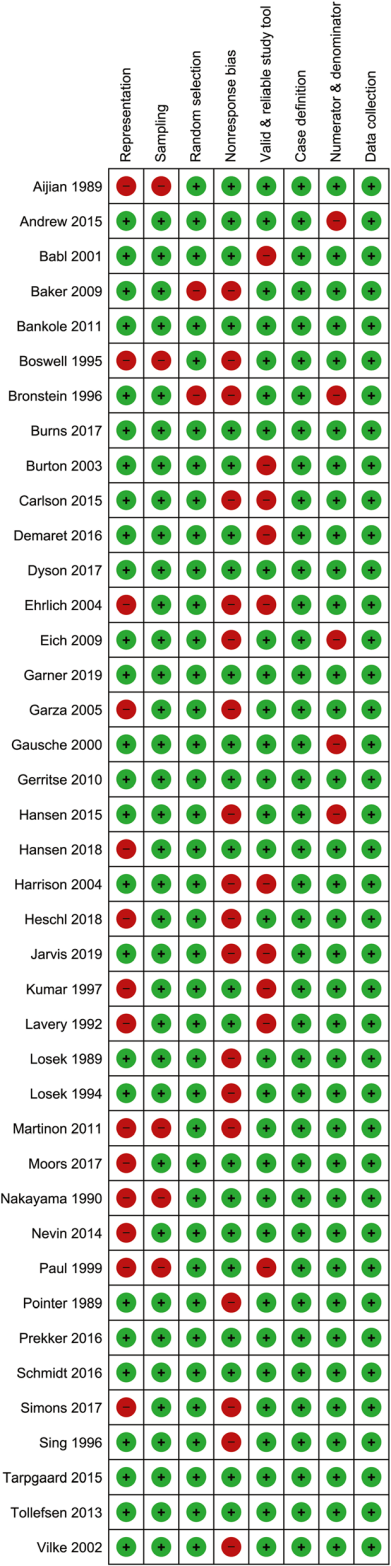
Review authors’ judgements about each risk of bias item for each included study. Low and high risk of bias are represented as green and red symbols, respectively

**Fig. 3:**
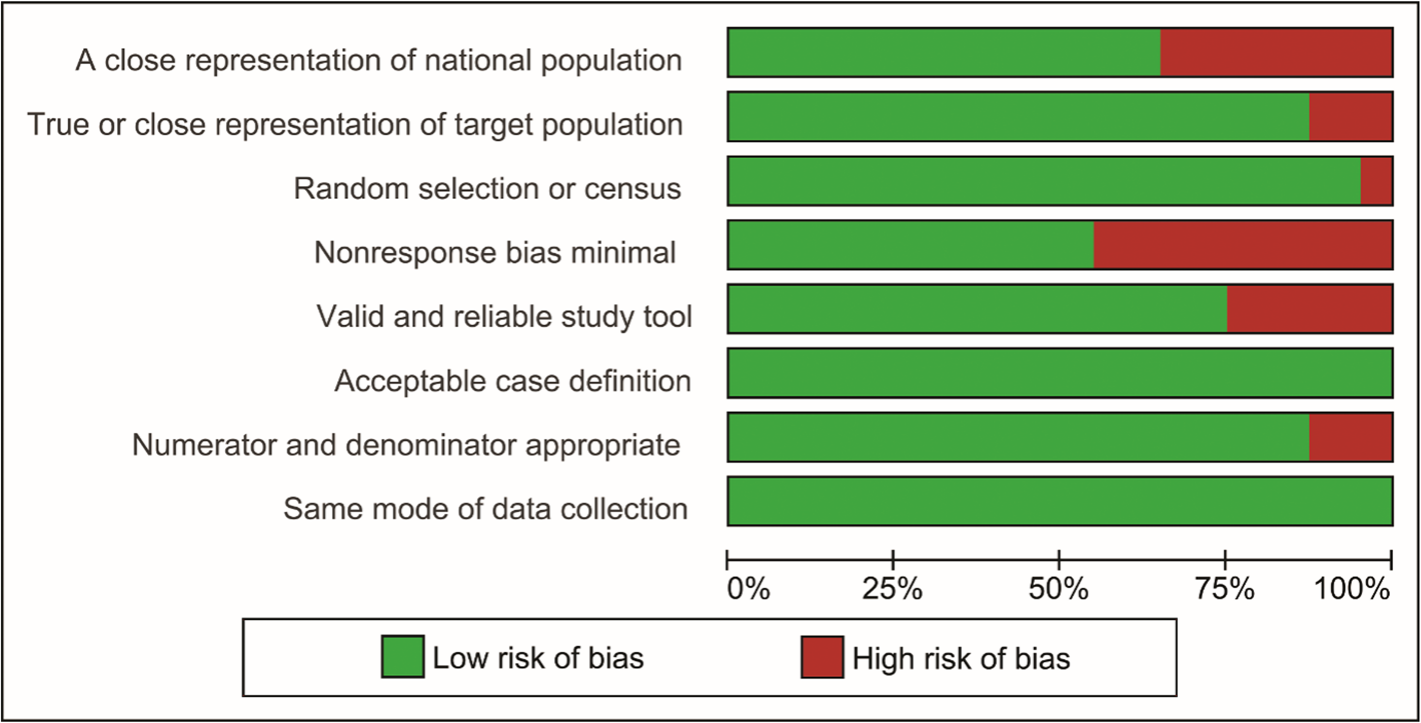
Review authors’ judgements about each risk of bias item presented as percentages across all included studies

None of the 40 studies were rated as high risk of bias. Eighteen studies (*n* = 7820) were rated moderate risk of bias, and 22 studies (*n* = 3747) had overall low risk of bias. Main reasons for biases for individual studies are shown in Fig. 2. Selection bias (items 1 to 3) was present in 16 studies (Fig. 3). Non-response bias (item 4) more than 20% was the most common type of bias affecting external validity (Fig. 3).

### Overall intubation and first-pass success rates

**Fig. 4:**
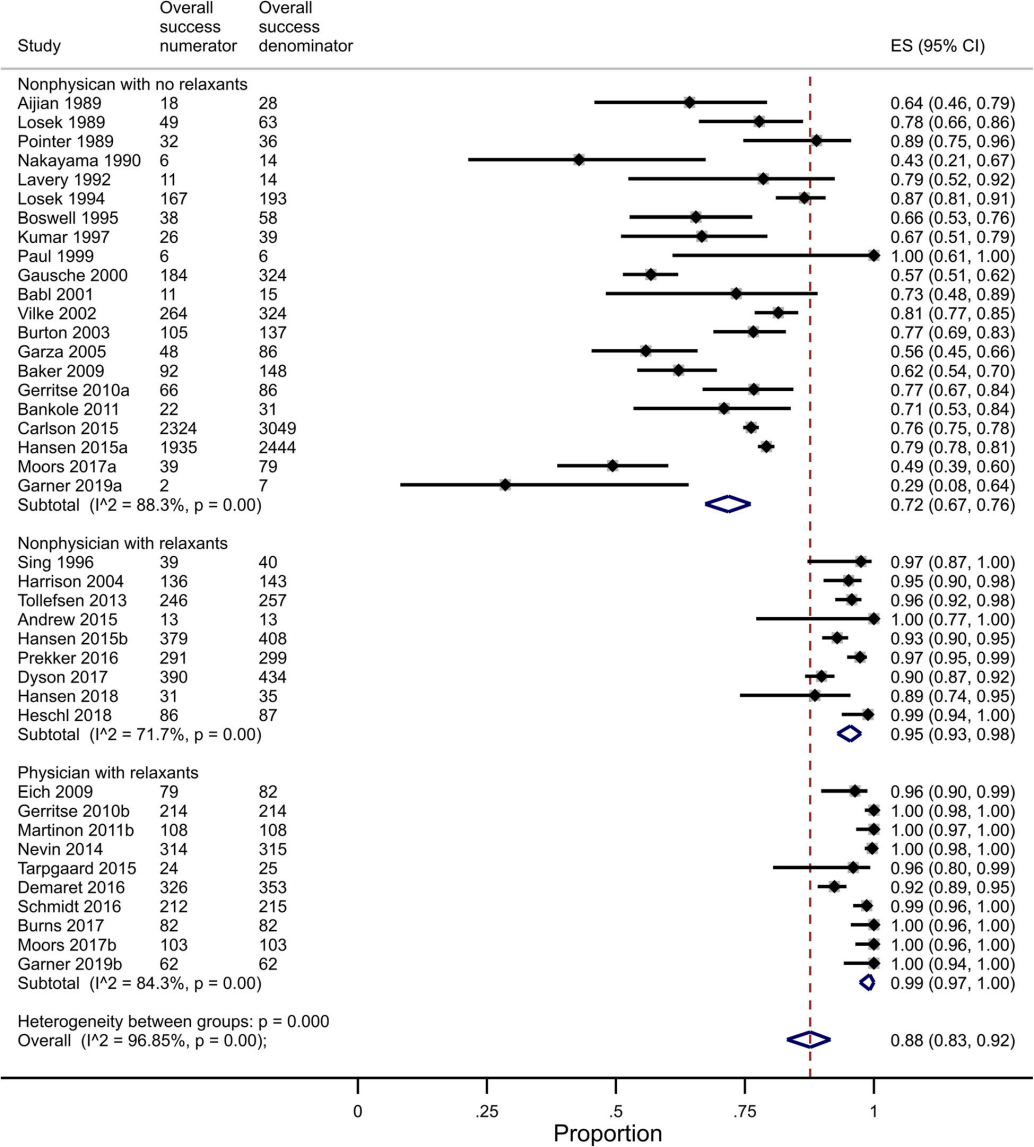
Pooled proportion for overall intubation success by clinical team groups

**Fig. 5:**
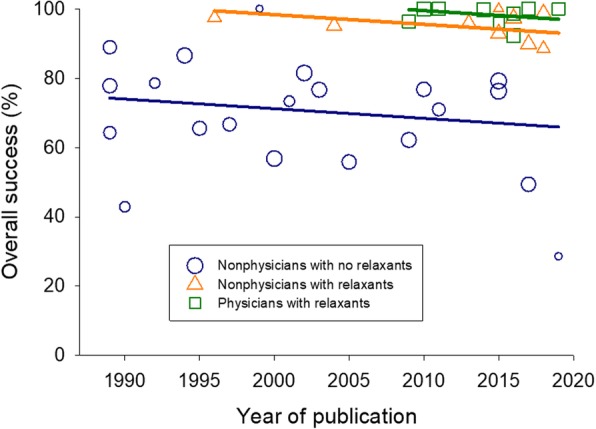
Meta-regression of overall intubation success over time (year of publication). Meta-regression lines are drawn for each clinical team group

Thirty-five studies (40 reports by a specific team type) in 10,456 children examined the overall success intubation rates, with most data coming from non-physician teams without relaxants (*n* = 7181). There was a significant intra-group heterogeneity (*I*^2^ > 72% for all three team composition groups) and a significant inter-group heterogeneity (*P* < 0.001), supporting the separate reporting of overall success intubation rates by subgroups. Non-physician teams without relaxants had lower overall success rates (72%, 95% CI 67–76%) than non-physician teams with relaxants (95%, 95% CI 93–98%) and physician teams (99%, 95% CI 97–100%) (Fig. 4). Differences in overall success rates by team composition were significant (*P* < 0.001) after adjusting for time (− 0.3%/year, 95% CI − 0.8%/year to 0.3%/year, *P* = 0.29) (Fig. 5).

The sensitivity analysis of 21 low risk of bias studies (24 team composition groups) in 3707 children showed similar results, with non-physician teams without relaxants having lower overall success rates (73%, 95% CI 64–81%) than non-physician teams with relaxants (96%, 95% CI 92–99%) and physician teams (99%, 95% CI 97–100%). Meta-regression in low risk of bias studies showed that team composition differences in overall success rates remained significant (*P* < 0.001) after adjusting for time (− 0.9%/year, 95% CI − 0.1%/year to − 1.6%/year, *P* = 0.04).

**Fig. 6:**
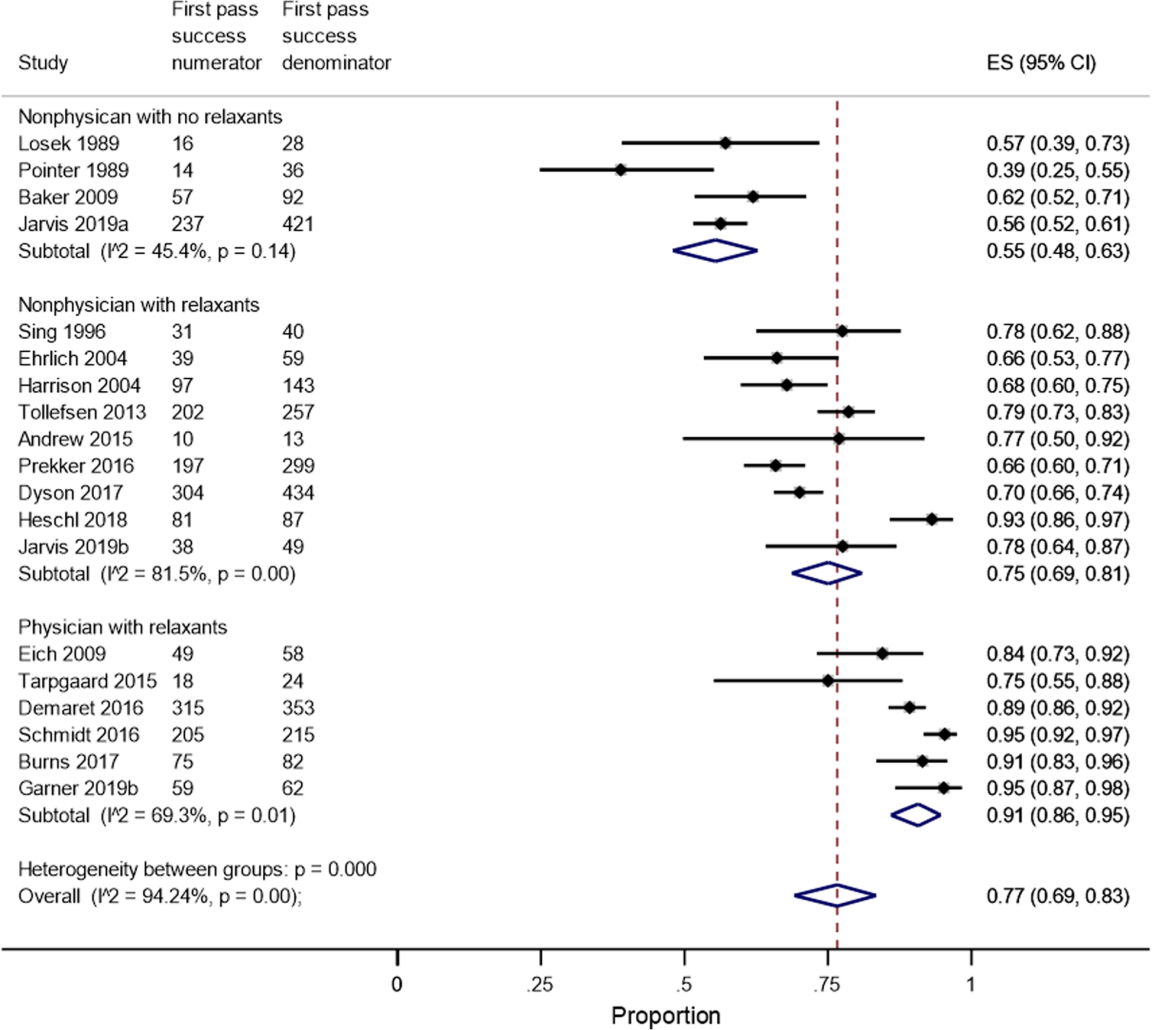
Pooled proportion for first-pass success by clinical team groups

Eighteen studies in 2752 children examined first-pass success rates (Fig. 6). There was a significant intra-group heterogeneity (*P* < 0.001) and a significant inter-group heterogeneity (*P* < 0.001), supporting the separate reporting of first-pass success intubation rates by subgroups. Physician teams had significantly higher first-pass success rates (91%, 95% CI 86–95%) than non-physician teams with relaxants (75%, 95% CI 69–81%) or without relaxants (55%, 95% CI 48–63%). Meta-regression was problematic as the degrees of freedom were less than 4 [15]. Sensitivity analysis in 12 low risk of bias studies (*n* = 1843) showed significant inter-group heterogeneity (*P* < 0.001), with physician teams associated with higher first-pass success rates (92%, 95% CI 87–96%) than non-physician teams with relaxants (73%, 95% CI 67–78%) or without relaxants (47%, 95% CI 35–59%).

### Adverse events

Most adverse events by team type showed large intra-group and inter-group heterogeneity, supporting the need to report individual team pooled estimates (Table 2). Sixteen studies, involving 1975 children, examined the overall intubation complication rate. The overall airway complication rate was lower in physician teams than in non-physicians with and without relaxants (Table 2). Physician teams were not associated with the occurrence of oesophageal intubations, aspirations, or the need for three or more multiple intubation attempts (*P* = 1.00, *P* = 0.27, *P* = 0.24, respectively).

**Table 2:** Summary of findings for adverse events by team composition

Outcome (total no. of studies)	Intubator group (total no. of children)	Pooled prevalence (%, 95% CI)	Intra-group heterogeneity (*I*^2^, *P* value)	Inter-group heterogeneity (*P* value)
Overall airway complications (16)	Non-physician without relaxants (536)	39 (28–51)	82.0%, *P* < 0.001	*P* < 0.001
	Non-physician with relaxants (667)	30 (23–38)	71.5%, *P* = 0.01	
	Physician with relaxants (772)	10 (3–22)	94.4%, *P* < 0.001	
Oesophageal intubations (23)	Non-physician without relaxants (3136)	4 (0–10)	92.3%, *P* < 0.001	*P* < 0.001
	Non-physician with relaxants (1563)	0 (0–1)	58.1%, *P* = 0.02	
	Physician with relaxants (815)	0 (0–0)	0%, *P* = 0.98	
Endobronchial intubations (13)	Non-physician without relaxants (311)	11 (6–17)	35.8%, *P* = 0.17	*P* = 0.15
	Non-physician with relaxants (610)	10 (4–19)	85.8%, *P* < 0.001	
	Physician with relaxants (260)	3 (0–11)	85.1%, *P* < 0.001	
	Pooled (1181)	7 (3–12)	86.2%, *P* < 0.001	
Hypoxia (6)	Non-physician with relaxants (183)	0 (0–1)	NR	*P* < 0.001
	Physician with relaxants (468)	7 (3–11)	94.4%, *P* < 0.001	
Aspiration (8)	Non-physician with relaxants (587)	12 (4–23)	90.6%, *P* < 0.001	*P* < 0.001
	Physician with relaxants (206)	0 (0–2)	0%, *P* = 0.47	
≥ 3 intubation attempts (13)	Non-physician without relaxants (96)	16 (6–28)	NR	*P* < 0.001
	Non-physician with relaxants (816)	6 (3–10)	69.7%, *P* = 0.01	
	Physician with relaxants (544)	1 (0–4)	75.6%, *P* = 0.01	

All outcome comparisons, except endobronchial intubation, had large inter-group heterogeneity (i.e. large variations in results between groups) preventing overall pooling of results. *NR* not reported as less than 3 groups, no intra-group heterogeneity reported

As there was no inter-group heterogeneity for endobronchial intubation (*P* = 0.15), the overall pooled estimated was 7% (95% CI 3–12%) (Table 2). However, Simons and colleagues’ study [44] appears to be an outlier (21%, 95% CI 10–37%) as there were exhaustive attempts to determine the endotracheal tube position after arrival in the emergency department. A post hoc sensitivity analysis, excluding Simons and colleagues’ study [44], showed that there was a significant inter-group heterogeneity (*P* < 0.001), with a pooled estimate for physician team decreasing to 0% (95% CI 0–2%).

Of the 40 studies included in this systematic review, only six [7, 10, 30, 37, 45, 46] (651 children) reported hypoxia after intubation. Much of the meta-analysis result was influenced by Martinon and colleagues’ study [37] (*n* = 296) that examined the effect of national guidelines on prehospital intubation in severely head-injured children. A post hoc sensitivity analysis, excluding Martinon and colleagues’ study [37], showed that there was a significant inter-group heterogeneity (*P* < 0.001), with a pooled estimate for physician team decreasing to 3% (95% CI 0–10%).

### Outcomes by clinical population

Seven studies were in trauma patients (*n* = 500) [21, 26, 34, 39–41, 44], six in arrested patients (*n* = 804) [5, 17, 25, 29, 33, 38], and three in head injury patients (*n* = 414) [6, 31, 37], and the remaining studies comprised a mixed population of all patients requiring airway management (*n* = 9849). There was no association between team composition and type of paediatric patients treated (*P* = 0.875). Non-physicians with or without relaxants had lower rates of overall intubation success in arrested patients compared with other patient populations (Table 3). The first-pass success rate and overall airway complication rate by patient population and team composition are shown in Table 3.

**Table 3:** Summary of findings for overall successful intubation, first-pass success, and overall airway complication rates by patient population and team composition

Outcome	Patient population	Intubator group (total no. of studies, total no. of participants)	Pooled prevalence (%, 95% CI)	Intra-group heterogeneity (*I*^2^, *P* value)	Inter-group heterogeneity (*P* value)
Overall success	Mixed	Non-physician without relaxants (12, 6826)	76 (71–80)	90.0%, *P* < 0.001	*P* < 0.001
	Mixed	Non-physician with relaxants (6, 1160)	96 (94–98)	38.5%, *P* = 0.15	
	Mixed	Physician with relaxants (7, 1033)	99 (95–100)	86.1%, *P* < 0.001	
	Trauma	Non-physician without relaxants (4, 92)	71 (50–89)	65.4%, *P* = 0.03	*P* < 0.001
	Trauma	Physician with relaxants (1, 315)	100 (98–100)	NR	
	Head injury	Non-physician without relaxants (1, 31)	71 (53–84)	NR	*P* < 0.001
	Head injury	Non-physician with relaxants (1, 87)	99 (94–100)	NR	
	Head injury	Physician with relaxants (1, 108)	100 (97–100)	NR	
	Arrested	Non-physician without relaxants (4, 232)	57 (49–64)	22.5%, *P* = 0.28	*P* < 0.001
	Arrested	Non-physician with relaxants (2, 469)	90 (87–93)	NR	
	Arrested	Physician with relaxants (1, 103)	100 (96–100)	NR	
First-pass success	Mixed	Non-physician without relaxants (4, 577)	55 (48–63)	45.4%, *P* = 0.14	*P* < 0.001
	Mixed	Non-physician with relaxants (6, 801)	73 (67–79)	63.3%, *P* = 0.02	
	Mixed	Physician with relaxants (6, 794)	91 (86–95)	69.3%, *P* = 0.01	
	Trauma	Non-physician with relaxants (1, 59)	66 (53–77)	NR	NA
	Head injury	Non-physician with relaxants (1, 87)	93 (86–97)	NR	NA
	Arrested	Non-physician with relaxants (1, 434)	70 (66–74)	NR	NA
Overall complications	Mixed	Non-physician without relaxants (5, 463)	32 (20–45)	83.7%, *P* < 0.001	*P* = 0.01
	Mixed	Non-physician with relaxants (3, 608)	31 (22–40)	NR	
	Mixed	Physician with relaxants (5, 476)	8 (1–21)	92.8%, *P* < 0.001	
	Trauma	Non-physician without relaxants (1, 14)	43 (21–67)	NR	*P* = 0.26
	Trauma	Non-physician with relaxants (1, 59)	27 (17–40)	NR	
	Trauma	Pooled (2, 73)	30 (19–41)	NR	
	Head injury	Non-physician without relaxants (1, 31)	68 (50–81)	NR	*P* < 0.001
	Head injury	Physician with relaxants (2, 296)*	18 (14–23)	NR	
	Arrested	Non-physician without relaxants (1, 28)	46 (30–64)	NR	NA

All outcome comparisons had large inter-group heterogeneity (i.e. large variations in results between groups) preventing overall pooling of results, except for overall airway complications in trauma patients. *NR* not reported as less than 3 groups, no intra-group heterogeneity reported; *NA* not applicable

*Martinon et al. [37] before and after national guidelines

## Discussion

To our knowledge, this is the first meta-analysis to compare prehospital intubation success and complication rates of different teams of intubator providers specifically in children. The success and complication rates for physician teams are better than non-physician teams either with or without muscle relaxants. Although reported clinical populations varied between studies, the success and complications rates followed the same pattern when population subgroup meta-analyses were performed. The overall success and first-pass success estimates were robust in the sensitivity analyses. Even after adjusting for the year of publication in the meta-regressions, team composition differences in the overall success estimate remained significant. The overall quality of evidence was graded as moderate to high after assessing for the presence of selection and non-response bias, measurement bias, and bias related to data analysis [12].

Two previous meta-analyses [12, 49] examining the success and complication rates by physician and non-physician teams regardless of patient age where both team types utilised relaxants demonstrated higher overall and first-pass success for physician teams compared with non-physician teams. Our review indicates that this is also observed in the paediatric subgroup. A possible contributor to higher success rates by physician teams is in-hospital exposure to paediatric intubation compensating for the rare requirement for this procedure in prehospital practice. All of the identified physician team studies utilised HEMS for at least some responses, and it may be that there is an additive effect from HEMS increasing team experience by allowing small numbers of clinicians to cover a larger population thereby concentrating exposure. As non-physician teams utilising relaxants have higher success rates when transported by HEMS compared with ground transport lends additional support to this theory.

Successful use of a clinical bundle to avoid peri-intubation hypoxia by a non-physician team utilising relaxants in non-arrested adults has been reported [50]. The bundle mandated intubation attempts be abandoned in favour of mask ventilation and urgent transport when pre-oxygenation failed to achieve a SpO_2_ of at least 94%. The complete bundle reduced peri-intubation hypoxia rates from 44.2 to 3.5% and suggests that avoiding prehospital intubation in hypoxic patients may minimise risk for teams with lower experience levels. This approach however also potentially denies intubation to patients with critical hypoxia who are arguably the most likely to benefit from early intubation. A focus on oxygenation rather than procedural success is suggested for future studies given this is the primary aim of all airway management. It is noteworthy that in our systematic review, only six studies could be identified that reported hypoxia as an outcome from 40 studies that met the inclusion criteria.

Caution is also needed in interpreting our meta-analysis subgroup analysis results as these are observational in nature. However, we believe that the results of the within-study comparisons of different team composition performances in four studies [8, 28, 32, 38], together with insights from our recent study [7], are credible and supportive of higher overall intubation success, first-pass success, and lower complication rates associated with physician teams. Our results were also robust when sensitivity analysis and meta-regressions were performed. The definition of paediatric age group varied between studies ranging from < 13 to < 19 years. Inclusion of a large proportion of teenage patients in a sample is unlikely to reflect the specific issues of paediatric airway management as the greatest difficulty and complication rates occur in smaller children.

Differences in airway training between studies and between team types are a possible explanation for the observed performance differences. As a major difference between physicians and non-physicians is the training programmes to which they have been exposed, it is intuitive to suggest that further training of non-physician teams may decrease or eliminate the observed differences. Reporting of airway training was too heterogeneous to support an analysis however. Some studies provided no description of training [19, 21, 24, 26, 39, 44, 45], and some reported pooled data from multiple agencies [3, 28, 32], whilst others described the studied teams simply as Advanced Life Support and/or Paediatric Advanced Life Support certified [5, 20, 33–35].

It is possible that there is variability between team types in willingness to report complications. Studies have demonstrated under-reporting of prehospital intubation complications by non-physician personnel [51] and physician teams [7] when documentation is compared with electronic monitor data. Similarly, under-reporting has been documented in the emergency department setting when video recordings of the resuscitation are reviewed [52]. We are not aware of any studies that compare the rates of under-reporting between team types however. Under-reporting is also likely to be affected by factors such as organisational and national cultures which may confound any difference by team type as well as the status of legal protection for disclosure of complications in the reporting jurisdiction. Ideally, future studies should report complications based on monitor data and/or video review.

## Conclusions

Our systematic review supports higher overall success and first-pass success with lower complication rates by teams incorporating physicians when intubating children in the prehospital environment. The results of the meta-analysis suggest that this applies regardless of non-physician team utilisation of neuromuscular blockade. Physician prehospital teams should be utilised wherever practicable for critically ill children requiring prehospital intubation.

## Supplementary information

**Additional file 1.**

## Data Availability

The datasets used and/or analysed during the current study are available from the corresponding author on reasonable request.
