# Supplementary material for: Success and complications by team composition for prehospital paediatric intubation: a systematic review and meta-analysis
Source: Crit Care. 2020 Apr 15;24:149. doi: 10.1186/s13054-020-02865-y (PMC7161251; doi:10.1186/s13054-020-02865-y)
Supplement: Supplementary file 1 — Additional file 1. [file 13054_2020_2865_MOESM1_ESM.docx]

**Supplementary Online Content**

**eAppendix.** Search strategy

*Medline MeSH search:*

1 Airway Management/

2 Intubation/

3 Pediatrics/

4 Child/

5 Emergency Medical Services/

6 1 or 2

7 3 or 4

8 5 and 6 and 7

*Medline Keyword search:*

1 Prehospital.mp.

2 out-of-hospital.mp.

3 pediatric.mp.

4 paediatric.mp.

5 intubation.mp.

6 Airway.mp.

7 1 or 2

8 3 or 4

9 5 or 6

10 7 and 8 and 9

*Embase MeSH search*

1 airway/

2 intubation/

3 pediatrics/

4 child/

5 emergency health service/

6 1 or 2

7 3 or 4

8 5 and 6 and 7

*Embase Keyword search*

1 Prehospital.mp.

2 out-of-hospital.mp.

3 pediatric.mp.

4 paediatric.mp.

5 Intubation.mp.

6 Airway.mp.

7 1 or 2

8 3 or 4

9 5 or 6

10 7 and 8 and 9

*CINAHL MeSH search*

1 (MH "Airway Management+")

2 (MH "Intubation+")

3 (MH "Pediatrics+")

4 (MH "Child+")

5 (MH "Emergency Medical Services+")

6 S1 OR S2

7 S3 OR S4

8 S6 AND S7

9 S5 AND S8

*CINAHL Keyword search*

1 Prehospital

2 out-of-hospital

3 pediatric

4 paediatric

5 intubation

6 airway

7 S1 OR S2

8 S3 OR S4

9 S5 OR S6

10 S7 AND S8 AND S9
